# Considerations on Electrochemical Technologies for Water Purification and Wastewater Treatment

**DOI:** 10.3390/ijerph20126140

**Published:** 2023-06-16

**Authors:** Josiel Martins Costa

**Affiliations:** School of Food Engineering (FEA), University of Campinas (UNICAMP), Campinas 13083-862, SP, Brazil; josiel@unicamp.br

Water scarcity and pollution are global issues caused by factors, such as population growth, industrialization, and the utilization of water resources [1]. Over the past few decades, the deterioration of ecosystems and the accumulation of synergistic effects have amplified negative environmental impacts, particularly within aquatic ecosystems [2]. In 2020, only 74% of the world’s population (approximately 5.8 billion people) had access to safe drinking water, leaving around 2 billion individuals without proper water services. In response, Target 6.1 of the United Nations Sustainable Development Goals aims to achieve universal and equitable access to safe and easily accessible drinking water by 2030 [3]. This target also seeks to assist developing countries in implementing water and sanitation initiatives and programs [4]. Additionally, Target 6.3 focuses on enhancing water quality, wastewater treatment, and safe reuse practices. As a result, there is a need for research and development of sustainable technologies that are both straightforward to construct and maintain, as well as which are specifically tailored for water treatment and recycling in rural or less developed regions. 

Anthropogenic activities, including domestic and industrial operations, generate wastewater. Its composition may include organic toxins, depending on the source of discharge. Industries, such as oil, food, textiles, pharmaceuticals, and construction, are major contributors to industrially generated wastewater [5,6,7]. Similarly, processes involving metals such as the electroplating industry generate metal-rich wastewater [8,9,10]. The concentration of components creates an environmental pollution crisis, which affects fauna and flora due to the increase in chemical oxygen demand (COD) [11]. Therefore, wastewater must undergo treatment using available technologies before being discharged into water bodies or soil.

Electrochemical technologies have proven to be effective in treating wastewater containing various components. These technologies offer advantages, such as cost-effectiveness, operational flexibility, and environmental friendliness [12,13]. Figure 1 illustrates the electrochemical approach, which encompasses multiple technologies that can be used individually or in combination with traditional methods to eliminate contaminants and produce freshwater that meets regulatory standards. The electrocoagulation mechanism involves applying an external voltage to the soluble anode, resulting in the production of cations. Colloidal contaminants in the water react with these cations, losing their stability and settling after coagulation. Electro-oxidation degrades and mineralizes organic pollutants through the generation of free radicals [14,15]. Furthermore, it can aid in the recovery of metallic complexes when combined with electrodeposition through the decomplexation–reduction process. Electroflotation treatment relies on the buoyancy of light organic contaminants and the generation of small bubbles [16]. Electrodialysis removes contaminants from wastewater by utilizing electrically driven membranes [17]. The separation of ions with different valence states occurs through monovalent perm-selective ion exchange membranes. Additionally, bipolar membranes generate OH^-^ and H^+^ ions on each side of the membrane via the electrolysis of water.

Capacitive deionization is a prominent technology used for water purification. When the electrodes are polarized in parallel, they guide ions towards the electric double layer on the porous surface of the electrodes, where the ions are temporarily immobilized [18]. In order to obtain deionized water, electrodesorption is employed to reverse or eliminate the electric field, releasing ions back into the solution and regenerating the saturated electrodes [19]. The electrodeionization process involves three steps: replacement of ions in the resin with a solution, application of an electric field to facilitate ion movement through the resin, and subsequent resin regeneration. Lastly, the electro-Fenton process utilizes an electrochemical reaction, where the H_2_O_2_ produced at the electrode reacts with Fe^2+^ to generate hydroxyl radicals [20]. This enables the oxidation and breakdown of recalcitrant pollutants into smaller molecules.

Drawing from a broad range of technologies discussed earlier, optimizing and modeling processes can lead to energy savings and the production of ultrapure water. Mass transfer mechanisms serve as foundations for mathematical simulations [21]. Moreover, linear programming can identify key parameters essential for equipment design and scale-up. It is anticipated that future electrochemical water purification technologies will operate and be constructed in an energy-efficient and affordable manner, bringing benefits to both the environment and society. Furthermore, wastewater contaminants possess considerable chemical energy that can be harnessed during degradation processes, leading to the simultaneous production of pollutants. By controlling electrode reactions and refining the micro-interface, pollutants can be selectively and efficiently separated and transformed. Another aspect involves the recovery of valuable metals from wastewater. Thus, combining electrochemical technologies with other approaches has demonstrated itself as an effective and promising strategy for sustainable water treatment and metal recovery.

## Figures and Tables

**Figure 1:**
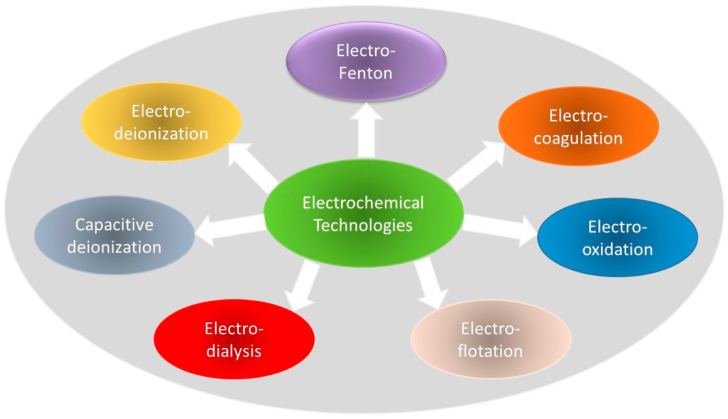
Electrochemical technologies for water and wastewater treatment.
